# Supplementary figures and images for: A comparative study of statistical methods for identifying differentially expressed genes in spatial transcriptomics
Source: PLoS Comput Biol. 2026 Feb 11;22(2):e1013956. doi: 10.1371/journal.pcbi.1013956 (PMC12912703; doi:10.1371/journal.pcbi.1013956)

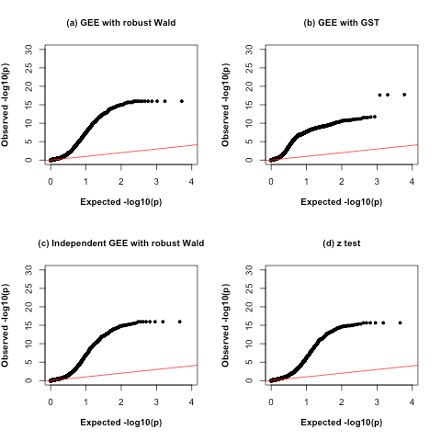

Supplement: S1 Fig — (PNG) [file pcbi.1013956.s001.png]

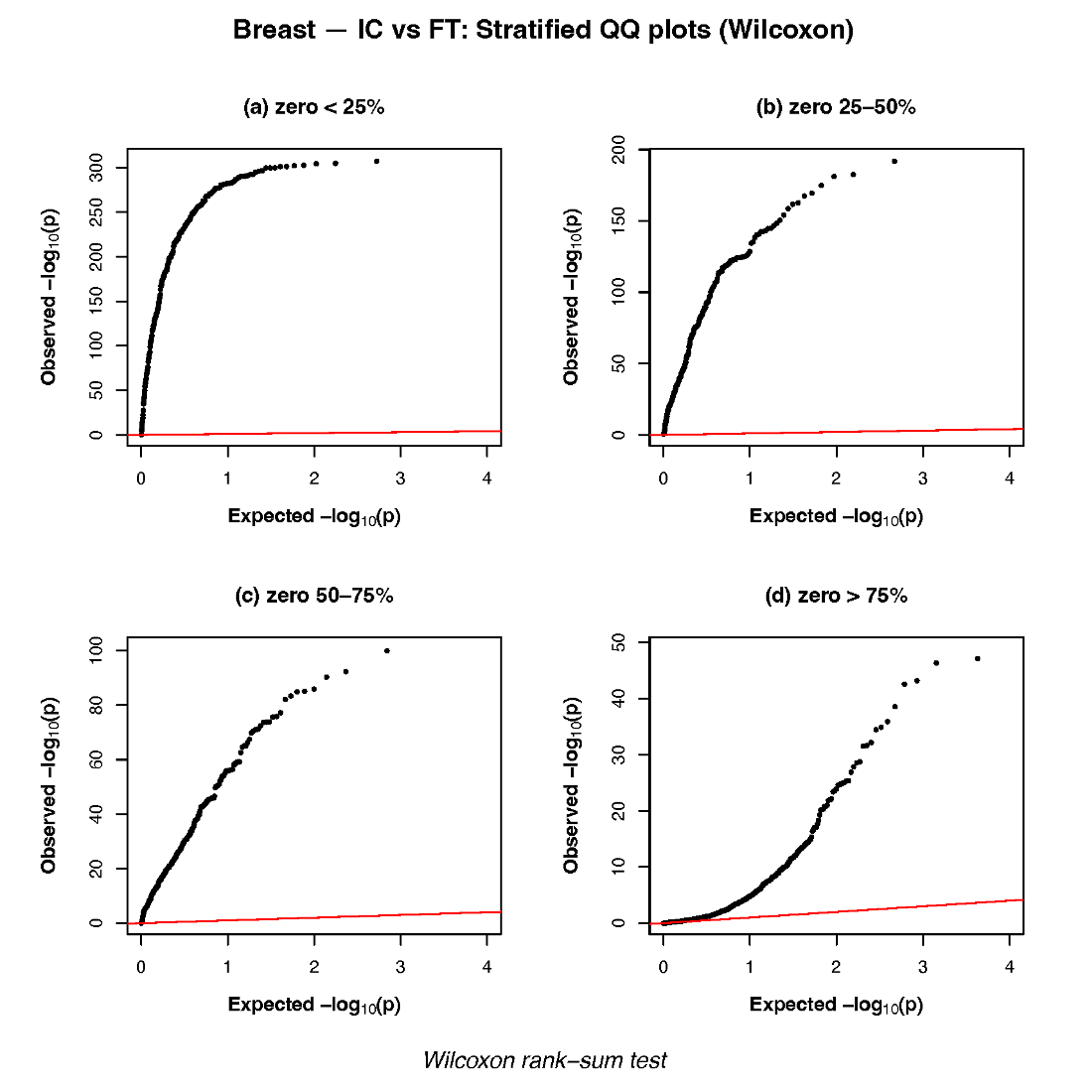

Supplement: S2 Fig — (PNG) [file pcbi.1013956.s002.png]

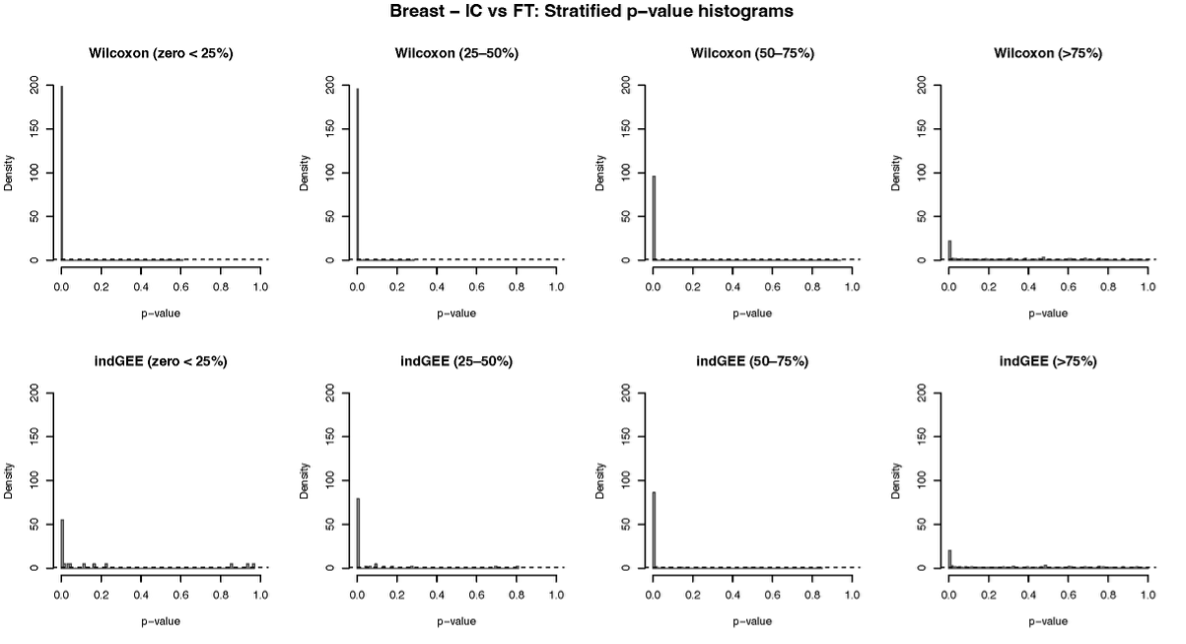

Supplement: S3 Fig — (PNG) [file pcbi.1013956.s003.png]

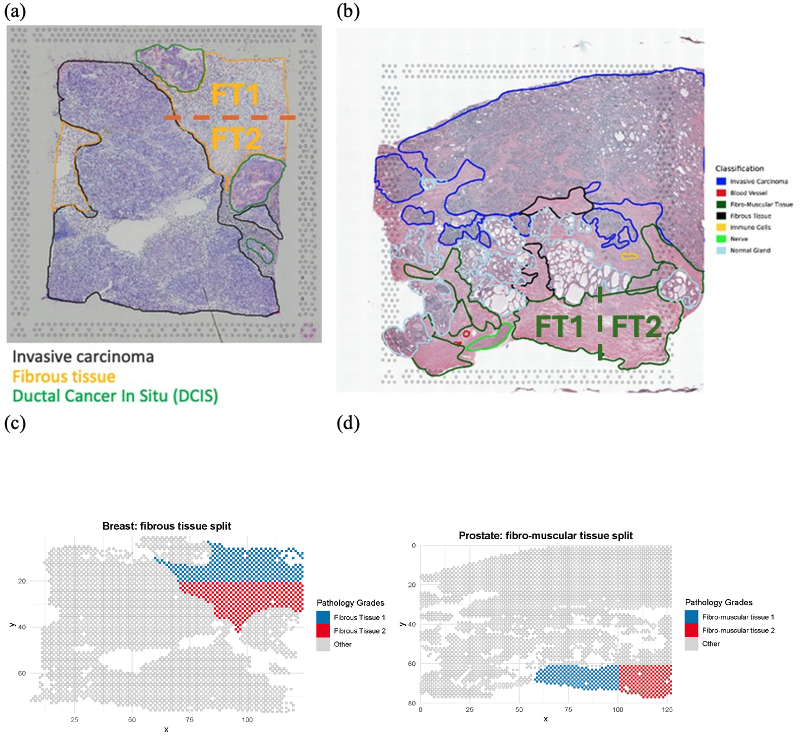

Supplement: S4 Fig — (PNG) [file pcbi.1013956.s004.png]

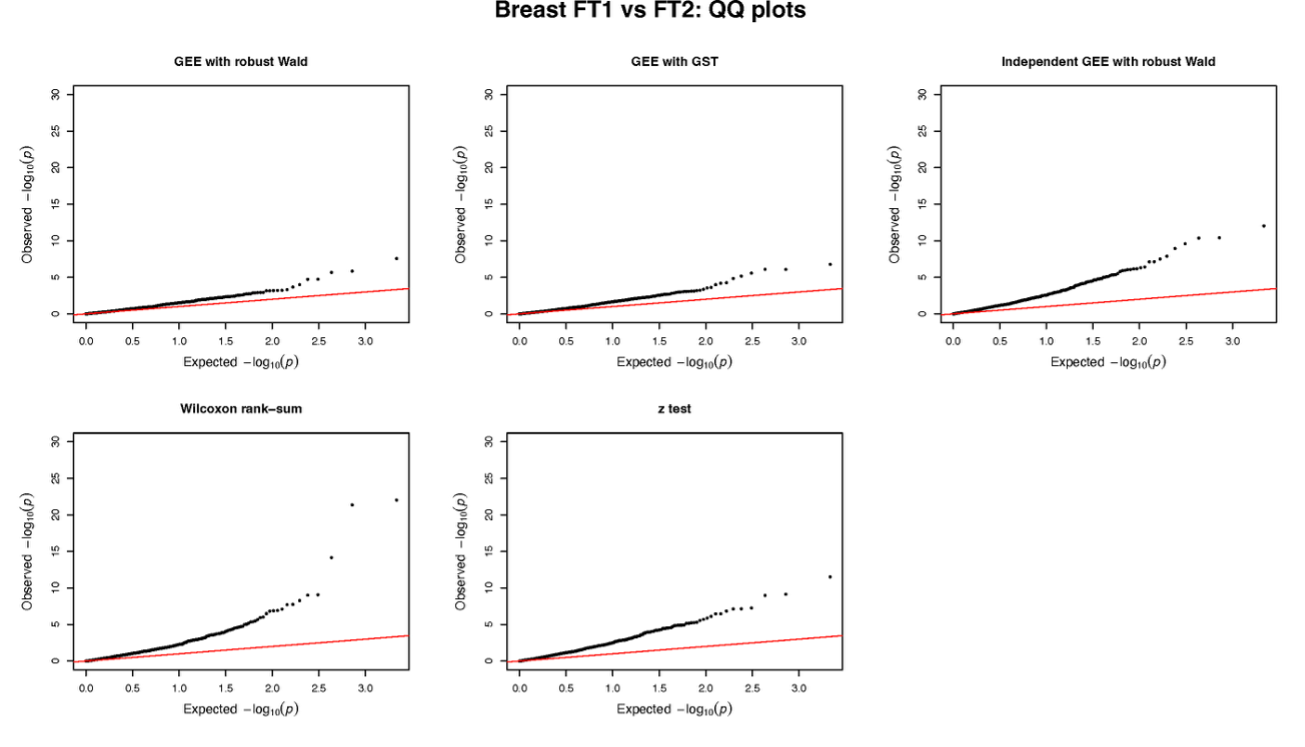

Supplement: S5 Fig — FT: fibrous tissue. (PNG) [file pcbi.1013956.s005.png]

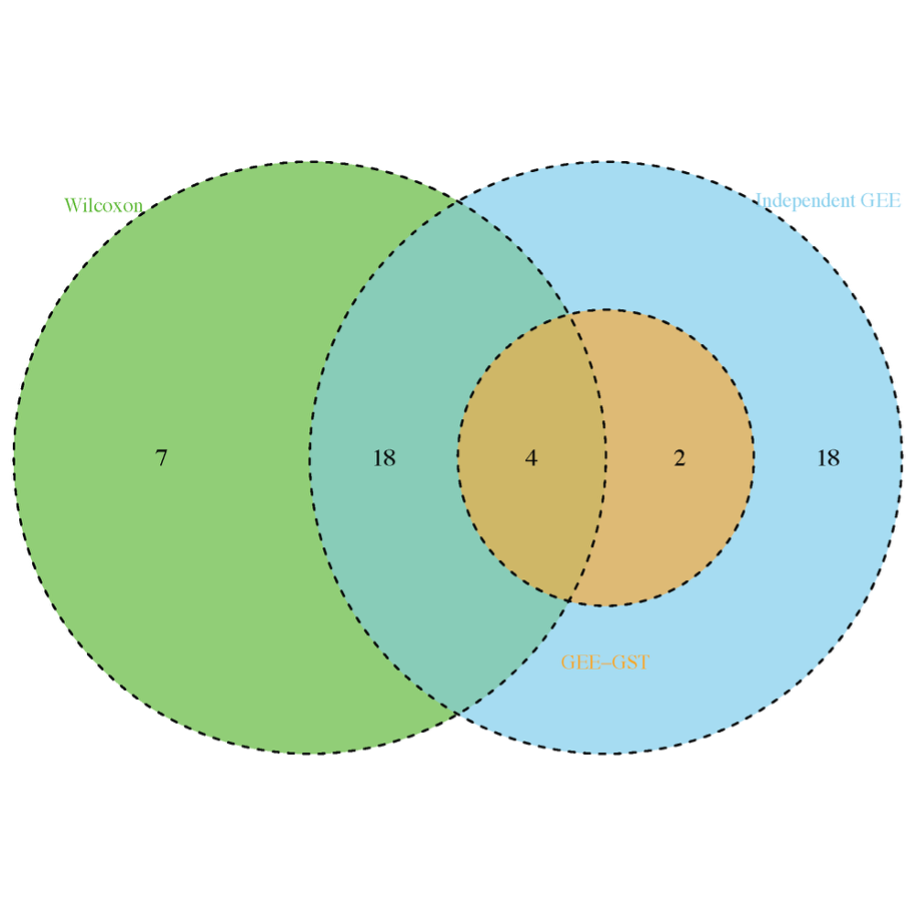

Supplement: S6 Fig — (PNG) [file pcbi.1013956.s006.png]

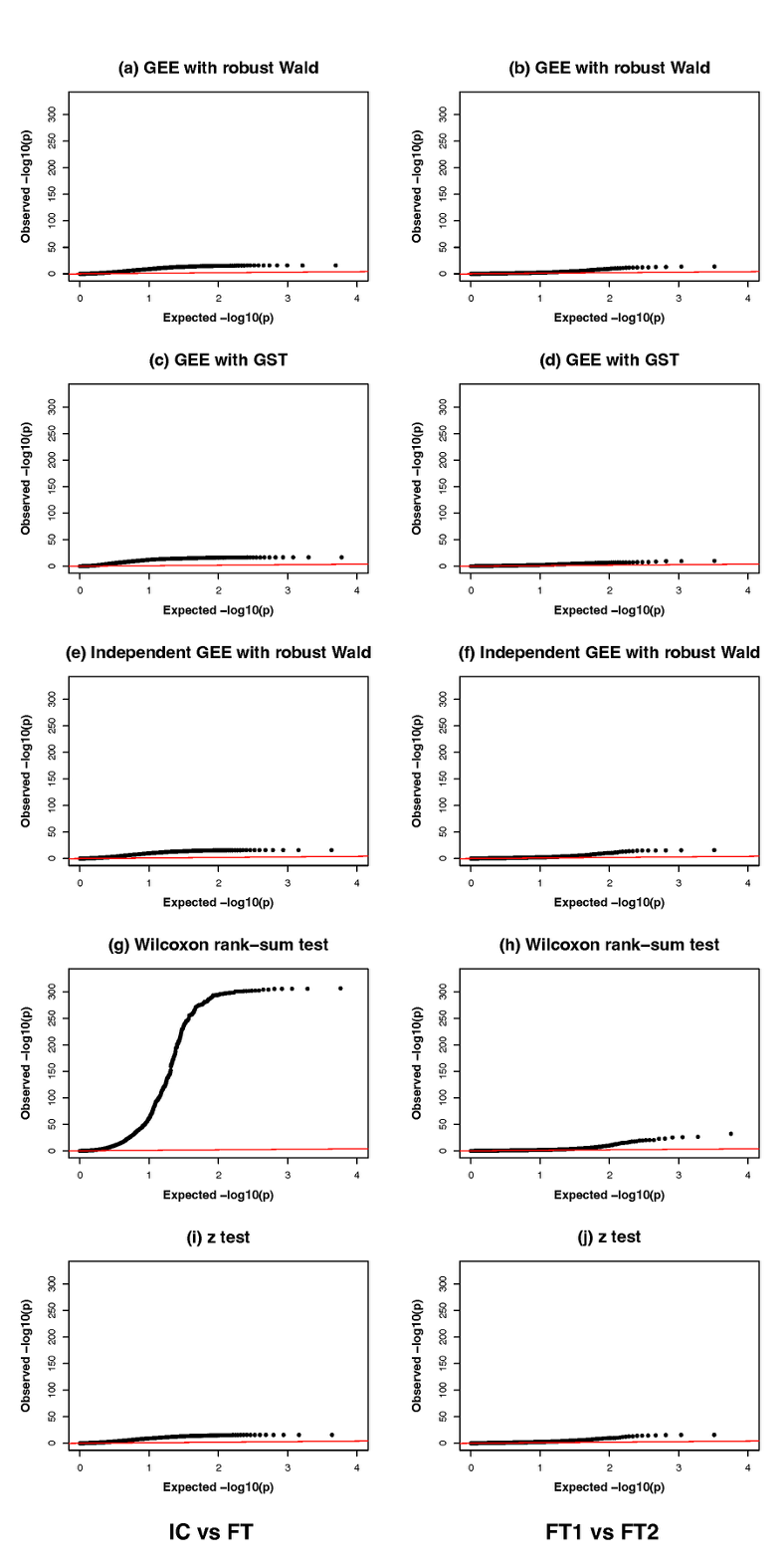

Supplement: S7 Fig — (PNG) [file pcbi.1013956.s007.png]

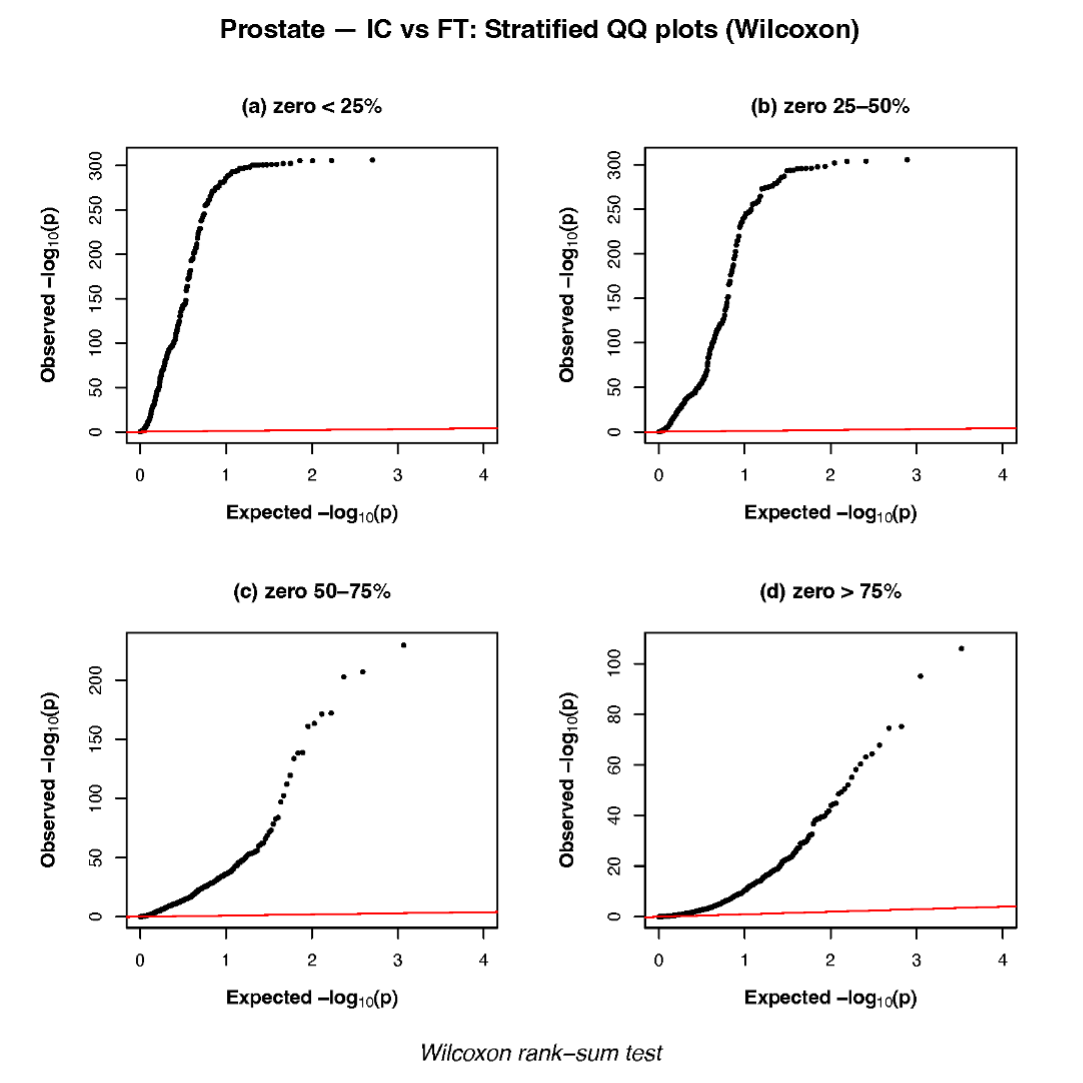

Supplement: S8 Fig — (PNG) [file pcbi.1013956.s008.png]

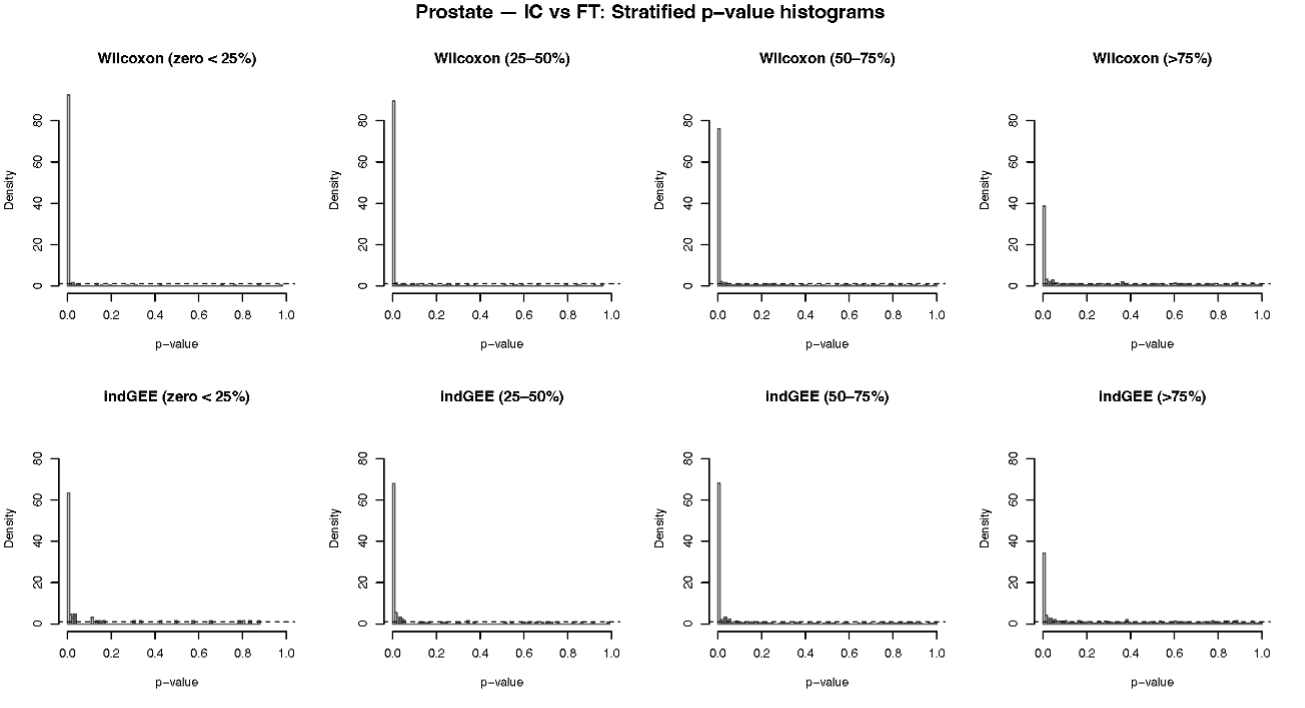

Supplement: S9 Fig — (PNG) [file pcbi.1013956.s009.png]

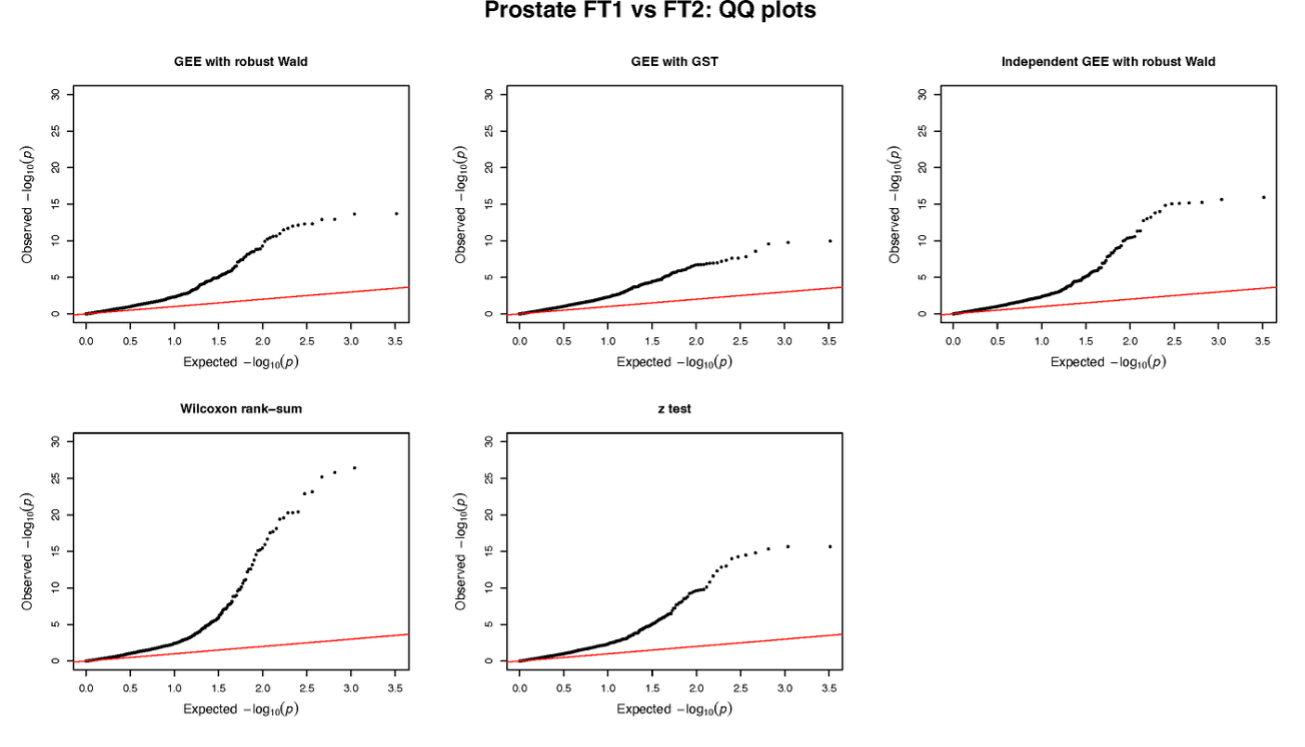

Supplement: S10 Fig — (PNG) [file pcbi.1013956.s010.png]

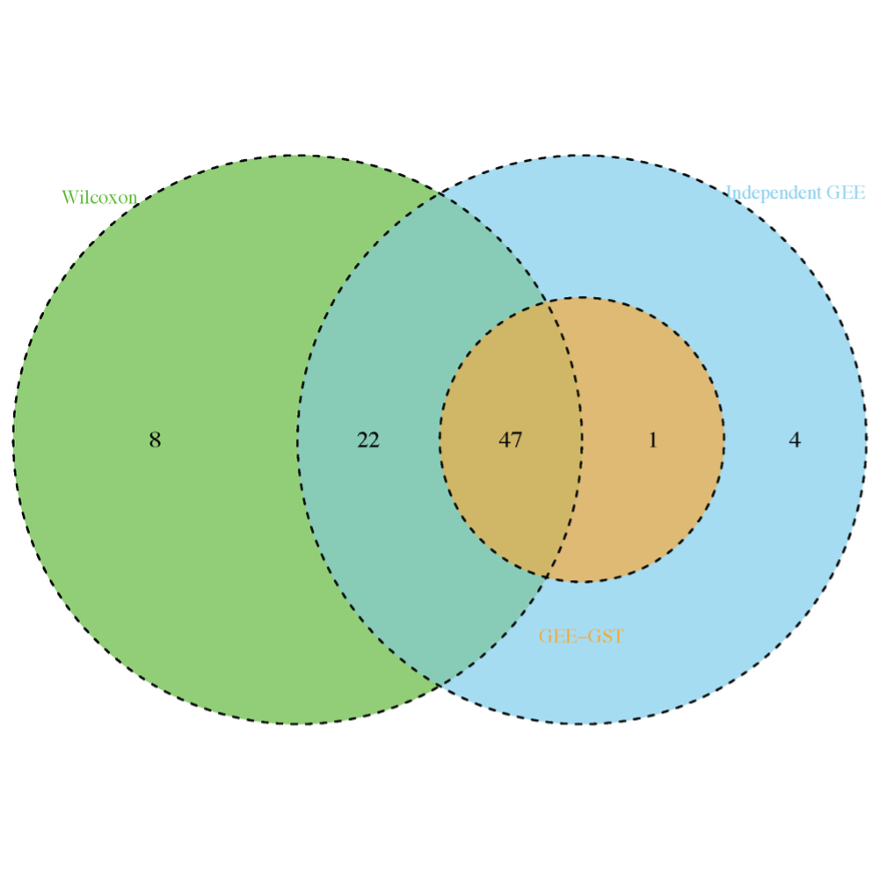

Supplement: S11 Fig — (PNG) [file pcbi.1013956.s011.png]

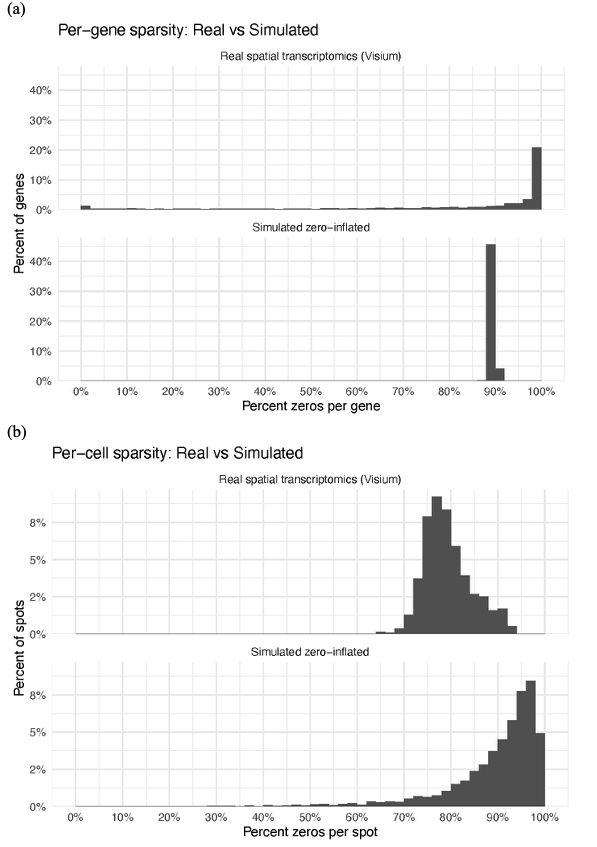

Supplement: S12 Fig — (PNG) [file pcbi.1013956.s012.png]
